# Saline Wastewater: Characteristics and Treatment Technologies

**DOI:** 10.3390/molecules28041622

**Published:** 2023-02-08

**Authors:** Jingtao Bi, Yingying Zhao

**Affiliations:** 1 School of Chemical Engineering and Technology, Hebei University of Technology, No. 8, Guangrong Road, Hongqiao District, Tianjin 300130, China; 2 Engineering Research Center of Seawater Utilization of Ministry of Education, No. 8, Guangrong Road, Hongqiao District, Tianjin 300130, China; 3 Hebei Collaborative Innovation Center of Modern Marine Chemical Technology, No. 8, Guangrong Road, Hongqiao District, Tianjin 300130, China; 4 Tianjin Key Laboratory of Chemical Process Safety, Tianjin 300130, China

The discharge of saline wastewater has significantly increased due to rapid urbanization and industrialization [1,2,3]. Saline wastewater contains substantial amounts of salt resources originating from various sources, such as NaCl from the coal chemical industry and Zn(II) from electroplating processes [4,5,6]. Despite the presence of these valuable resources, due to the uncertainty in the handling cost and profit, saline wastewater is generally transported to the wastewater treatment plant or evaporated into solid salt wastes in most industries. Nevertheless, such a treatment would not only be a waste of resources but also place a significant treatment burden on downstream facilities.

Similar to the nineteenth century “clouds” over the dynamical theory of heat and light, two small, puzzling “clouds” remained on the horizon of saline wastewater treatment. The first one comes into the contradiction between a continuously complex salt matrix and perusing for simplified utilization of wastes, and the second one is the challenge for sustainable optimization of energy consumption and carbon emissions.

In response to these “clouds”, two major concepts have emerged in the quest for efficient and sustainable saline wastewater treatment: zero discharge and carbon emission reduction [7,8]. To achieve zero discharge, a variety of methods have been proposed to extract resources from saline wastewater, including nanofiltration, reverse osmosis, evaporation and crystallization [9,10,11,12]. During these processes, energy conservation and integrated technology were extensively utilized to decrease energy consumption and reduce carbon emissions. In the impending future, these two concepts might serve as the linchpins and hold the potential to evolve into the “Quantum Mechanics” and “Theory of Relativity” of saline wastewater treatment.

This Special Issue, entitled “Saline Wastewater: Characteristics and Treatment Technologies”, focuses on the resource recycling and carbon emission reductions in saline wastewater treatment, including innovative technologies and the related fundamental characterizations of saline wastewater. During the production of this Special Issue, we aimed to highlight our inspirations and contribute to the development of sustainable solutions for this critical environmental issue:

(1) There are huge amounts of coexisting contaminants in some saline wastewater, such as phenols and heavy metals [13,14]. This circumstance directly induces the extracted liquid/solid salts might have unsatisfied purity and contain hazardous materials, which is the main reason for the extremely low price of the extracted salts. It is imperative to develop high-purity extraction and secondary refining techniques in order to mitigate these challenges.

(2) With the continuous progress of new materials, equipment and methods, their integration with saline wastewater treatment presents a significant challenge. The integration has the potential to bring forth novel technologies and play a crucial role in the realization of zero discharge and carbon emission reduction across the entire treatment process [15]. While the current focus of integration efforts is on new methods and equipment, the integration of saline wastewater treatment with sustainable and cost-effective advanced materials has the potential to become a highly sought-after area of research.

(3) The cost and carbon emissions are the main considerations in the treatment of saline wastewater. To ensure its viability, the value of the products, state subsidies and potential benefits should offset the costs associated with capital construction and energy consumption. With the development of treatment techniques and the utilization of renewable energy, it is important to conduct systematic life cycle economic analysis and carbon emission assessments to reassess the treatment of saline wastewater.

Finally, we hope the published manuscripts in this Special Issue will give prospects to handle the two small “clouds” in this area. Good luck!
